# Retraction: Exploring Pharmacological and Non-Pharmacological Approaches to Managing Hypertension During Pregnancy: A Systematic Review

**DOI:** 10.7759/cureus.r172

**Published:** 2025-04-01

**Authors:** Saeeda Bano, Shahida Husain Tarar, Precious D Atung, Abdullah Shehryar, Abdur Rehman, Ahmad Irshad, Oluwatobiloba T Ogungbemi, Nahal Ijaz, Maryam Nour, Hafsah Abdirahiim Maalim

**Affiliations:** 1 Obstetrics and Gynecology, Sahiwal Medical College, Sahiwal, PAK; 2 Obstetrics and Gynecology, Aziz Bhatti Shaheed Teaching Hospital, Gujrat, PAK; 3 Obstetrics and Gynecology, Shenyang Medical College, Kaduna, NGA; 4 Internal Medicine, Allama Iqbal Medical College, Lahore, PAK; 5 Surgery, Mayo Hospital, Lahore, PAK; 6 General Surgery, Mayo Hospital, Lahore, PAK; 7 Internal Medicine, Caucasus International University, Tbilisi, GEO; 8 Medicine and Surgery, Avicenna Hospital, Lahore, PAK; 9 Family Medicine, Emergency Medicine, Internal Medicine, Psychiatry, John F. Kennedy University School of Medicine, Williemstad, CUW; 10 Emergency Medicine, Henry Ford Health System, Detroit, USA; 11 Internal Medicine, Omdurman Islamic University, Khartoum, SDN

The Editors-in-Chief have retracted this article. An investigation by the journal found evidence of authorship manipulation. The Editors-in-Chief therefore no longer have confidence in the provenance and reliability of the article contents. The authors agree with the decision to retract.

